# Real-Time Structural Health Monitoring and Damage Identification Using Frequency Response Functions along with Finite Element Model Updating Technique

**DOI:** 10.3390/s22124546

**Published:** 2022-06-16

**Authors:** Tarunpreet Singh, Shankar Sehgal, Chander Prakash, Saurav Dixit

**Affiliations:** 1 University Institute of Engineering and Technology, Panjab University, Chandigarh 160014, India; tarun0512@pu.ac.in; 2 School of Mechanical Engineering, Lovely Professional University, Phagwara 144411, India; chander.21503@lpu.co.in; 3 Peter the Great St. Petersburg Polytechnic University, 195251 Saint Petersburg, Russia; 4 Division of Research & Innovation, Uttaranchal University, Dehradun 248007, India

**Keywords:** finite element, model updating, structural dynamics, structural health monitoring

## Abstract

Throughout service, damage can arise in the structure of buildings; hence, their dynamic testing becomes essential to verify that such buildings possess sufficient strength to withstand disturbances, particularly in the event of an earthquake. Dynamic testing, being uneconomical, requires proof of concept; for this, a model of a structure can be dynamically tested, and the results are used to update its finite element model. This can be used for damage detection in the prototype and aids in predicting its behavior during an earthquake. In this instance, a wireless MEMS accelerometer was used, which can measure the vibration signals emanating from the building and transfer these signals to a remote workstation. The base of the structure is excited using a shaking table to induce an earthquake-like situation. Four natural frequencies have been considered and six different types of damage conditions have been identified in this work. For each damage condition, the experimental responses are measured and the finite element model is updated using the Berman and Nagy method. It is seen that the updated models can predict the dynamic responses of the building accurately. Thus, depending on these responses, the damage condition can be identified by using the updated finite element models.

## 1. Introduction

Over the course of time, several instances of damage can occur in an engineering structure. Condition monitoring and damage identification of engineered structures are vital for the longevity of the structures, as well as in reducing the overall maintenance cost [1]. Technological advancements in the discipline of structural health monitoring (SHM) have been growing rapidly in the last two decades. SHM has been employed in the monitoring of almost every engineered structure, from rotating machinery [2,3] to bridges and buildings [4,5]. This technique includes various damage detection methods, namely vibration-based sensors, embedded sensors, acoustic emissions, lamb wave method, and comparative vacuum monitoring [6]. Humans and the environment largely influence the health of these structures and, with time, damage can arise in these structures which can be attributed to aging, inadequate service conditions, and erroneous manufacturing. 

Cawley et al. [7] proposed studying the natural frequencies of a structure to identify and locate damage. They also studied the growth of damage using the proposed technique [8]. Kessler et al. [9] compared the dynamic responses of the control and damaged specimens, and postulated that the reduction in the frequency response was attributed to the presence of damage. 

Many techniques of SHM have been comprehensively researched and used for damage detection, and it has been established that vibration-based damage detection is a well-recognized and uncomplicated technique to study the dynamic characteristics of an engineered structure. Various researchers have utilized a wide variety of methods to analyze them. The dynamic characteristics include the damping ratio, natural frequencies, and mode shapes, which are a function of mass and stiffness distribution of the structure. Any variation in the mass and stiffness distribution, due to any external influence, will result in a deviation of the dynamic characteristics from the control structure. A direct correlation has been acknowledged between damage and a decrease in the natural frequency of the system.

Intelligent monitoring techniques have been used for damage localization and quantification in numerous operational bridges [10], buildings [11], and aerospace structures [12], by identifying the deviation from the optimal working conditions and determining remaining life. Mishra [13] systematically reviewed the advantages of combining test data with machine learning for structural health monitoring and damage prognosis, to ensure the longevity of heritage buildings. Gopinath et al. [14] also reviewed the various long-term and short-term techniques for damage identification and localization in heritage structures. Numerous researchers have also focused their study on the condition monitoring of heritage structures [15,16]. 

Kessler et al. [9] compared the dynamic responses of control and damaged specimens, and postulated that the reduction in the frequency response was attributed to damage. The loss of stiffness assisted in the detection and localization of the damage [17]. Fan et al. [18] compared various vibration-based methods for damage detection in composite materials. It was proposed that the natural frequency-based methods could localize the damages, whereas curvature and mode shape-based methods called for optimization algorithms for localization. Yan et al. [19] also proposed the effectiveness of combining conventional vibrational theory with other methods, such as artificial intelligence, control theory, and signal processing, etc., to increase the accuracy of vibration-based structural health monitoring techniques.

Alavi et al. [20] employed finite element modelling and a probabilistic neural network approach. They interpreted data from a wireless sensor and identified damage in a simply supported beam in the complex case of bridge gusset plate. Tran-Ngoc et al. [21] proposed a hybrid metaheuristic algorithm approach to overcome the limitations of genetic algorithms, and used an improved cuckoo search technique for solving optimization issues and damage detection in a bridge structure. Hsu et al. [22] studied the vibration response of a building structure using a wireless sensor for damage localization and quantification in a building structure. Feng et al. [23] compared transmissibility function and cross-correlation analysis for damage detection in a tunnel structure, and proposed using both approaches for validation of the results.

The major novelty of this work is applying the combined use of the modified Berman and Nagy direct method of finite element model updating with wireless sensors in real-time health monitoring along with subsequent damage identification under the event of an earthquake or seismic disturbance by studying the change in frequency response functions (FRFs), which has not been explored previously under the aforementioned combinations.

This paper will focus on designing and developing a real-time monitoring and subsequent damage identification technique, using the structural frequency response and finite element model updating by exciting a scaled-down two-story building model on a vibrating table. This proposed technique will not only apply to buildings, but to every structure for which a finite element model can be prepared and a vibrational analysis performed.

## 2. Analytical Methodology

### 2.1. Finite Element Method (FEM)

FEM is the most functional and acknowledged method used to analyze the performance of the building model and for finding the approximate solutions for field value problems in engineering [24,25]. The FEM model of the two-story building was generated using 34 2D frame elements with two translational degrees of freedom in the x and y directions, and one rotational degree of freedom, to model in-plane dynamics.

The finite element modelling of the structure can be subcategorized into three categories: pre-processing, processing, and post-processing.

#### 2.1.1. Pre-Processing

In this section, the input file created by the user defines the material properties, such as E, I, ρ, A, and L. The model structure, i.e., the coordinates of each element in the frame and its connectivity with other elements, is established. The degree of freedom of the nodes and initial matrices for an element are described by the user for processing in MATLAB.

#### 2.1.2. Processing

In the processing phase, the input given by the user is processed by MATLAB to fabricate the global mass and stiffness matrices, and boundary conditions are applied and solved for producing eigenvalues and eigenvectors. Symmetric global stiffness and mass matrices of size n produce n eigenvalues λ and corresponding eigenvectors *ϕ*, which satisfies equation [26].

$$\left[\bar{K}\right]\lambda_{i}=\lambda_{i}\left[\bar{M}\right]\phi_{i}$$

#### 2.1.3. Post-Processing

In this phase, the eigenvalues and the eigenvectors are utilized for producing the natural frequencies and mode shapes of the building frame under free-vibration conditions. The following operation is performed on the eigenvalue matrix for obtaining the natural frequency in rad/sec or Hz.

$$\left[\omega \right]=\sqrt{\left[\lambda \right]}$$

$$\left[f\right]=\frac{\left[\omega \right]}{2\pi }$$

The omega matrix [ω] will provide the natural frequency along its diagonal in rad/sec and [f] will give the natural frequency of the model in Hz.

### 2.2. Modal Analysis

**Figure 1:**
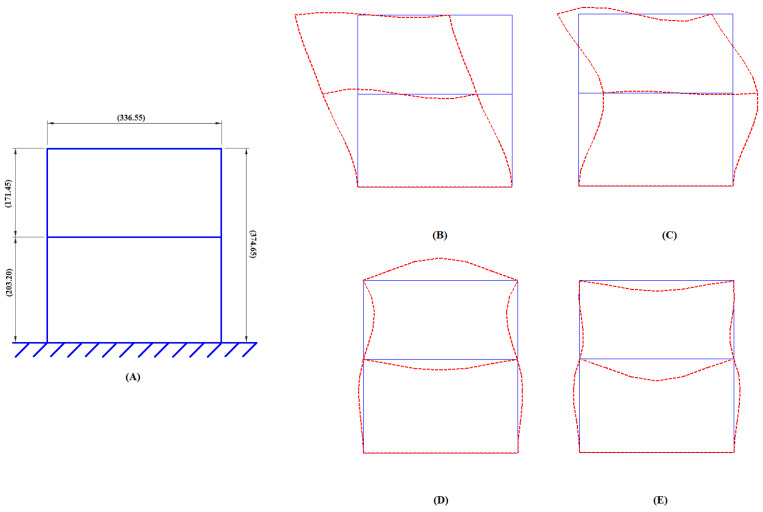
(**A**) Undeformed frame structure (all dimensions are in mm), (**B**–**E**) first four eigenmodes.

Asymmetric global stiffness and mass matrices were assembled, which produced eigenvalues and corresponding eigenvectors for yielding the natural frequencies and mode shapes of the building frame under free-vibration conditions. The first 4 natural frequencies of the analytical building model are listed in Table 1. Figure 1 illustrates the undeformed structure and the first 4 eigenmodes of the building model plotted in MATLAB.

### 2.3. Finite Element Model Updating

Inaccuracies in FE models can be attributed to errors such as: false material properties of constant values, inferior quality mesh, improper modeling of complex shapes and joints, and simplification and rounding off in computation [27]. The technique used to correct or update the analytical model (so that it can predict the dynamic responses accurately consistent with the experimental result) is known as finite model updating (FEMU) [28]. This technique can be broadly categorized into two categories: direct methods, such as the Baruch and Bar-ltzhack [29] and Berman and Nagy [30] methods, and indirect methods, such as the sensitivity method [28,31]. FEMU techniques have been reviewed [27,32,33] and the results show that FEMU should be combined with structural health monitoring for updating vibrating-based FE models for damage localization and quantification, for the maintenance of the structure and the prognosis of remaining life [34,35]. 

In the Berman and Nagy method, both the mass and stiffness matrices were updated using the eigenvector matrix *ϕ* that was updated as shown

$$\phi =\phi_{m}{\left[\phi_{m}^{T}M_{a}\phi_{m}\right]}^{\frac{-1}{2}}$$

$$\bar{M_{a}}=\phi_{m}^{T}M_{a}\phi_{m}$$

$$M_{u}=M_{a}+M_{a}\phi_{m}\bar{M_{a}^{-1}}\left(I-\bar{M_{a}}\right)\bar{M_{a}^{-1}}\phi_{m}^{T}M_{a}$$

and

$$K_{u}=K_{a}-K_{a}\phi \phi^{T}M_{a}+M_{a}\phi \phi^{T}K_{a}+M_{a}\phi \phi^{T}K_{a}\phi \phi^{T}M_{a}+M_{a}\phi \lambda \phi^{T}M_{a}$$

In this case, *ϕ* = corrected eigenvector matrix, *ϕ_m_* = experimental eigenvector matrix.

*M_a_* = analytical mass matrix, *M_u_* = updated mass matrix.

*K_a_* = analytical stiffness matrix, *K_u_* = updated mass matrix.

In this study, the Berman and Nagy method was modified by updating the eigenvector matrix *ϕ* using the structure’s frequency response.

## 3. Experimental Methodology

**Figure 2:**
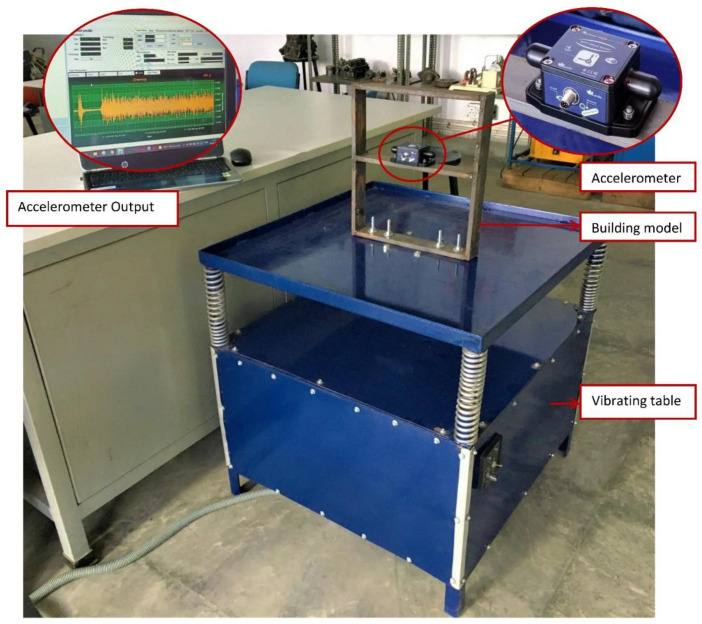
Experimental Setup.

Mild steel was selected as the workpiece for preparing the building model and was securely mounted on the vibrating table. The vibrating table provided continuous and constant vibration stimuli which acted as an excitation mechanism for the modal testing of the structure. The vibrational analysis of the building model was performed by employing a wireless MEMS accelerometer sensor. The acceleration measured by the sensor in the 3 axes is in the form of voltage and converted to a value of ‘g’. The entire experimental setup is shown in Figure 2.

**Figure 3:**
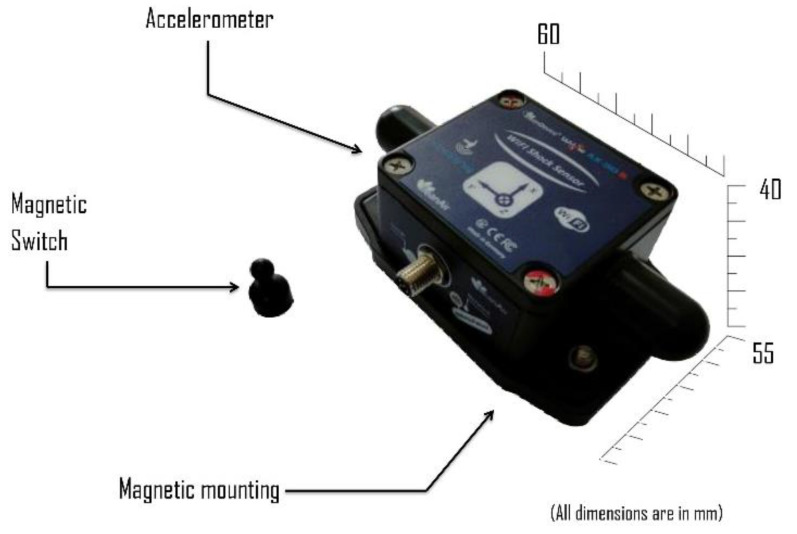
BeanDevice Wilow AX-3DS, MEMS Accelerometer.

A BeanDevice Wilow AX-3DS was employed for measuring the vibrations of the two-story building model. A BeanDevice Wilow AX-3DS is a micro-electromechanical system IoT based sensor, shown in Figure 3. It is an ultra-low-power, Wi-Fi enabled, tri-axial accelerometer dedicated to condition monitoring, vibrational analysis, and structural health monitoring. It has a measuring range of ±16 g with a maximum sampling of 1.6 k samples per second per axis. It integrates a 2.4 GHz antenna diversity, which provides a maximum wireless range of 200 m and a sensor frequency response (−3 dB) of DC to 800 Hz.

## 4. Experimental Results

Once the true experimental setup was established and the accelerometer was wirelessly connected to the personal computer, the vibrating table was operated to supply a constant and continuous excitation frequency to the building model at 100 to 400 rpm in 50 rpm incremental steps. The acceleration response was measured and then transformed from time-domain signal to frequency-domain signal for obtaining the natural frequencies of the model. 

The experimental results from the accelerometer sensor for the undamaged setup were exercised to update the FE model of the two-story building structure using the Berman and Nagy method, which improved the FE model to predict dynamic responses accurately. 

**Figure 4:**
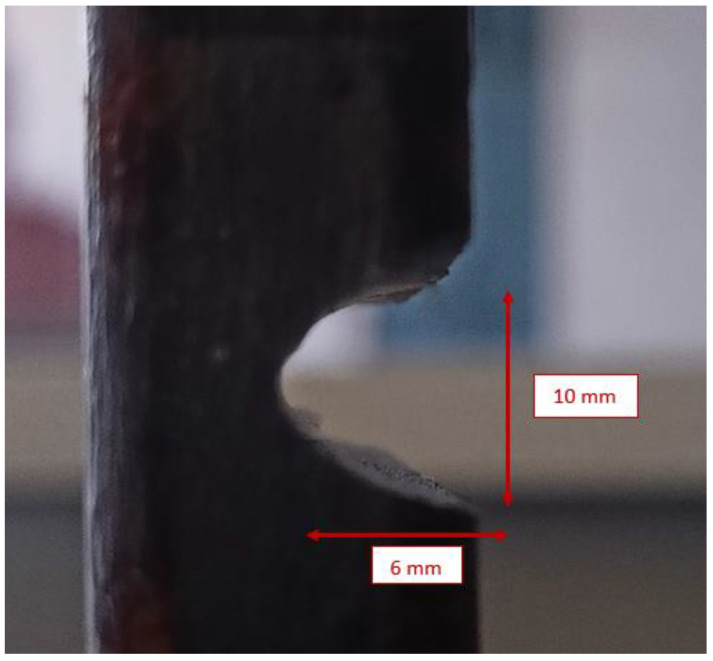
A sample of damage introduced to the building model.

A sample of damage introduced to the building model has been shown in Figure 4 and the seven conditions of the building are summarized in Table 2. The damage has dimensions of 10 × 6 mm^2^ and was introduced using a cutting tool. The seven conditions included one undamaged condition and six damaged conditions of the building model, and the dynamic response of the building was acquired using the accelerometer.

Utilizing the experimental results, a universal analytical model for the two-story building model can be generated. The natural frequencies of the first four modes of vibration are given in Table 3, which is true for all the excitation frequencies.

## 5. Discussion

**Figure 5:**
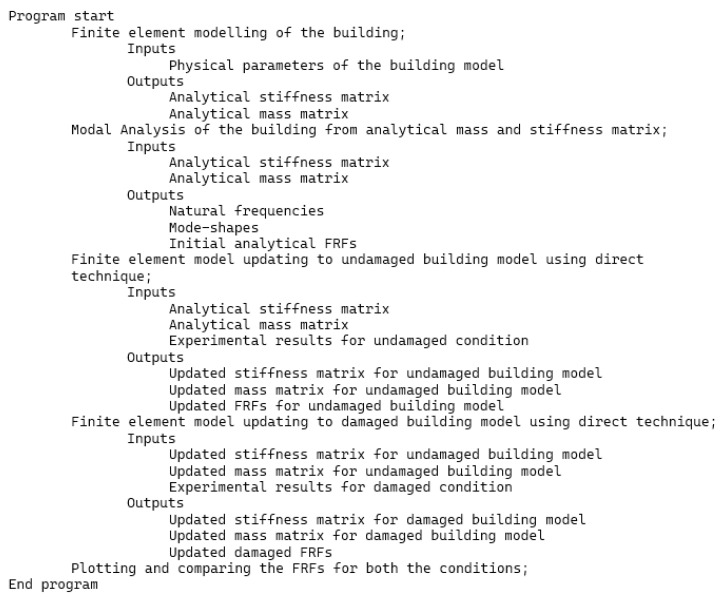
Pseudo code for extracting and plotting FRFs.

**Figure 6:**
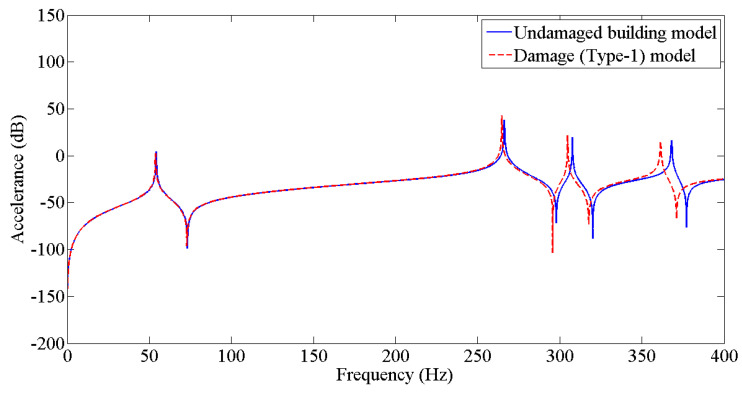
Prediction of structural dynamic behavior of building model under undamaged and damaged (Type−1) conditions through FRFs.

**Figure 7:**
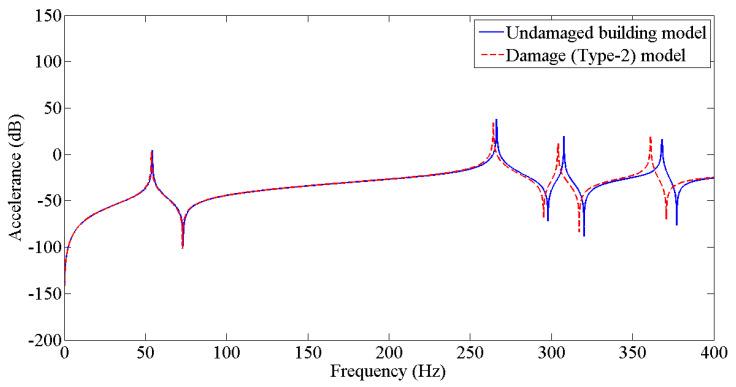
Prediction of structural dynamic behavior of building model under undamaged and damaged (Type−2) conditions through FRFs.

**Figure 8:**
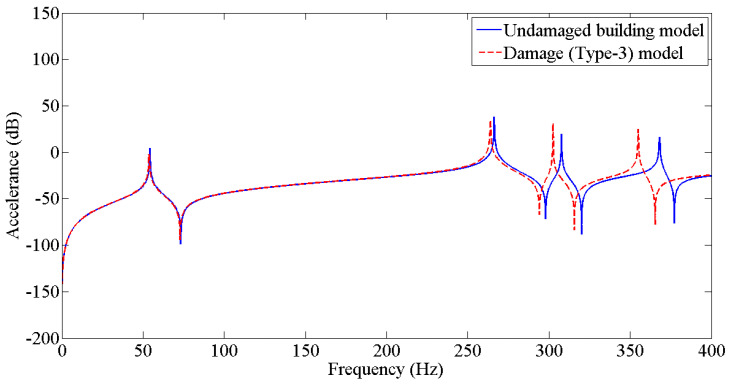
Prediction of structural dynamic behavior of building model under undamaged and damaged (Type−3) conditions through FRFs.

**Figure 9:**
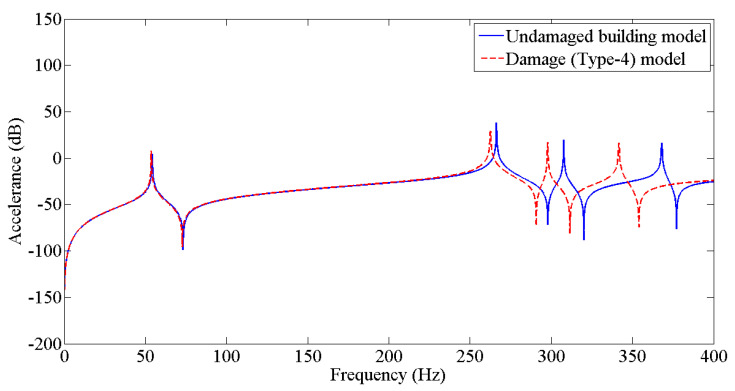
Prediction of structural dynamic behavior of building model under undamaged and damaged (Type−4) conditions through FRFs.

**Figure 10:**
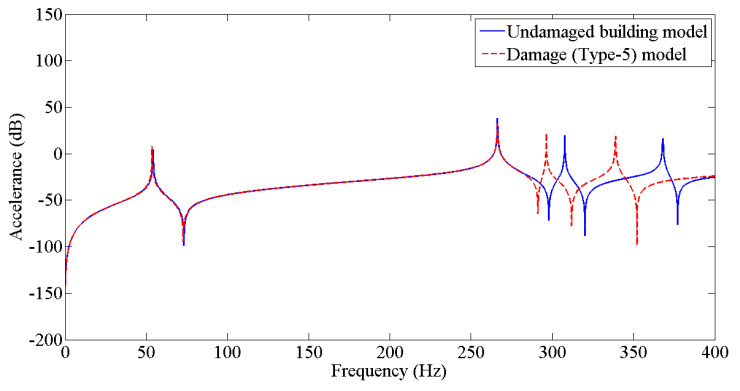
Prediction of structural dynamic behavior of building model under undamaged and damaged (Type−5) conditions through FRFs.

**Figure 11:**
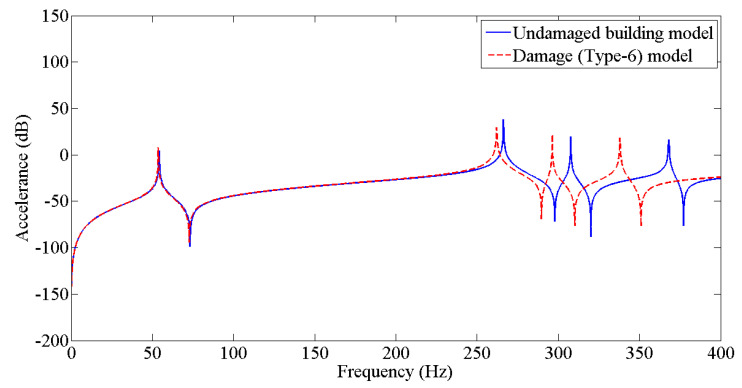
Prediction of structural dynamic behavior of building model under undamaged and damaged (Type−6) conditions through FRFs.

The results from Table 3 were employed to update the undamaged FE model to the various damaged conditions of the two-story building structure using the Berman and Nagy method. The pseudo code for plotting and comparing FRFs is discussed in Figure 5. Figure 6, Figure 7, Figure 8, Figure 9, Figure 10 and Figure 11 predict the structural dynamic behavior of the building model under undamaged and damaged conditions through FRFs for a better comparative study. The frequency response function, generally known as FRF, is a complex mathematical relationship between the input signal and the output signal for the system [36]. If the measured response is in the form displacement, then the corresponding FRF is called a receptance (admittance, dynamic compliance, or dynamic flexibility) FRF. Otherwise, velocity or acceleration response signals can be used to produce mobility or accelerance FRF, respectively. Accelerance FRF is also sometimes written as inertance FRF. The FRF can similarly be defined as the vibration response of a location owing to excitation at that location or any other location in the system. 

As discussed previously, dynamic responses are the function of stiffness and mass distribution of the structure. Variation in these, due to any external influence, will result in deviation of the dynamic response from the control structure; this was established after studying the dynamic response of the building following the introduction of damage conditions.

After analyzing the dynamic responses and FRFs of the system, it can be established that with the introduction of damage along the column, the natural frequencies of the system decrease. Furthermore, the magnitude of reduction in the natural frequency is directly proportional to the mode of vibration, i.e., the higher the mode of vibration, the larger the reduction in the frequency will be. It can also be postulated that the magnitude of reduction in the frequency of mode of vibration, in the case of a building structure due to damage, depends on three main factors: elevation of the damage from the base of the structure, extent of damage, and the presence of damage in the vicinity of that damage condition. 

**Figure 12:**
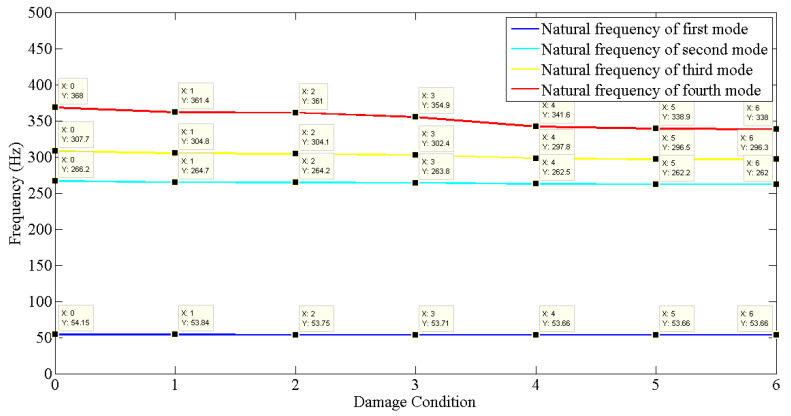
Reduction in frequencies of first four modes of vibration.

The magnitude of reduction is directly proportional to the elevation of the damage from the base of the structure and the extent of damage. It is inversely proportional to the presence of damage in the vicinity of that damage. Table 4 displays the elevation of each type of damage condition introduced to the building model. Figure 12 exhibits the reduction in the natural frequency of the first four modes of vibration for respective damage conditions.

As analyzed in the case of Type−1 and Type−2 damage conditions, the reduction in the frequency of a mode is lesser than that for Type−3 damage because of its elevation. The highest reduction in frequency can be seen in the case of Type−4 damage conditions, owing to its altitude and zero damages in its vicinity. Type−5 and Type−6 damage conditions are also present at a higher level of elevation, but the Type−4 damage condition already exists, due to which reduction in the frequency decreases. Initially, when smaller damage was created on the building model, the reduction in magnitude was found to be insignificant.

It is also evident from the experimental results that the natural frequency of the modes of vibration from the lower end tries to gradually attain a constant value, following which they are unaffected by the introduction of new damage conditions in the case of the building structure.

## 6. Conclusions

In this research, a scaled-down version of a two-story building model was developed experimentally and a finite element model was generated analytically. The experimental setup of the building model was subjected to vibratory stimuli from a shaking table at different frequencies. Modal testing was performed using wireless sensors in combination with the finite element model updating method of Berman and Nagy, so that the updated analytical model can predict the experimental dynamic response values accurately. Several damage conditions were introduced to the columns of the building model and the dynamic responses were scrutinized to analyze the magnitude of reduction in natural frequency and the factors influencing it. It was realized that the magnitude of reduction is directly proportional to the elevation of the damage from the base of the structure and extent of damage condition, and inversely proportional to the presence of other damages in the vicinity of that damage. It is also apparent that the natural frequency of the first mode of vibration gradually tries to attain a stagnant value, following which it is unaffected by the introduction of new damage conditions in the case of the building structure.

## Figures and Tables

**Table 1:** Analytical FEM based first 4 natural frequencies in Hertz.

Mode Number	1	2	3	4
Analytical natural frequency (Hz)	90.357	325.91	381.84	455.27

**Table 2:** Damage conditions of the building model (All dimensions are in mm).

Sr No.	Condition	Image	Description
1	Undamaged	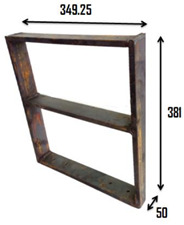	The two-story building model was made from MS flats of 50 mm width and 12 mm thickness. The dimensions (mm) of the scaled-down building model are shown in the image.
2	Damaged (Type−1)	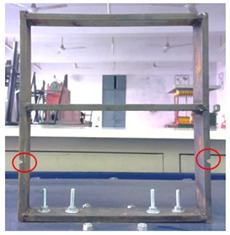	The damage condition (Type−1) was introduced to the walls of the first-floor of the building model, as shown, to study its dynamic responses. The damage was established using a cutting tool to cut slots of 10 × 6 mm^2^ along the width of the columns of the model at a height of 104.78 mm from the base.
3	Damaged (Type−2)	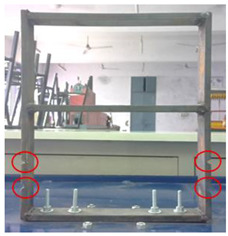	The damage condition (Type−2) was introduced to the walls of the first-floor of the building model, as shown, to study its dynamic responses. The damage was established using a cutting tool to cut slots of 10 × 6 mm^2^ along the width of the columns of the model at a height of 58.74 mm from the base.
4	Damaged (Type−3)	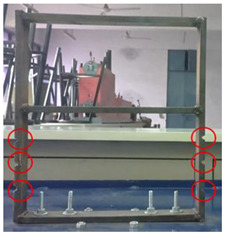	The damage condition (Type−3) was introduced to the walls of the first-floor of the building model, as shown, to study its dynamic responses. The damage was established using a cutting tool to cut slots of 10 × 6 mm^2^ along the width of the columns of the model at a height of 150.81 mm from the base.
5	Damaged (Type−4)	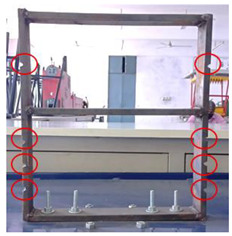	The damage condition (Type−4) was introduced to the walls of the second-floor of the building model, as shown, to study its dynamic responses. The damage was established using a cutting tool to cut slots of 10 × 6 mm^2^ along the width of the columns of the model at a height of 288.93 mm from the base.
6	Damaged (Type−5)	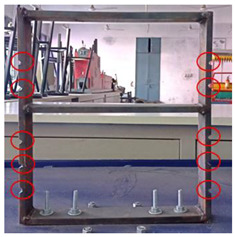	The damage condition (Type−5) was introduced to the walls of the second floor of the building model, as shown, to study its dynamic responses. The damage was established using a cutting tool to cut slots of 10 × 6 mm^2^ along the width of the columns of the model at a height of 249.24 mm from the base.
7	Damaged (Type−6)	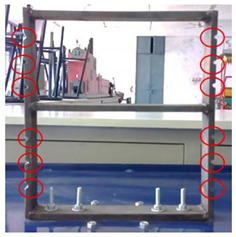	The damage condition (Type−6) was introduced to the walls of the second-floor of the building model, as shown, to study its dynamic responses. The damage was established using a cutting tool to cut slots of 10 × 6 mm^2^ along the width of the columns of the model at a height of 328.62 mm from the base.

**Table 3:** Updated natural frequencies (Hz) of undamaged and damaged analytical models.

Mode No.	Natural Frequency (Hz) of the Building Model						
	Un- Damaged	Damaged					
		Type−1	Type−2	Type−3	Type−4	Type−5	Type−6
1.	54.15	53.84	53.75	53.71	53.66	53.66	53.66
2.	266.24	264.69	264.24	263.84	262.53	262.16	262.04
3.	307.67	304.80	304.09	302.40	297.81	296.51	296.33
4.	368.04	361.36	361.01	354.89	341.64	338.89	337.99

**Table 4:** Elevation (mm) of all damage conditions.

Damaged Condition	Type−1	Type−2	Type−3	Type−4	Type−5	Type−6
Elevation (mm)	104.78	58.74	150.81	288.93	249.24	328.62
